# 2-[Anilino(phen­yl)meth­yl]cyclo­heptan­one

**DOI:** 10.1107/S1600536812050659

**Published:** 2012-12-19

**Authors:** Bagher Eftekhari-Sis, Sahar Mohajer, Maryam Zirak, Sakineh Mozaffarnia, Orhan Büyükgüngör

**Affiliations:** a Department of Chemistry, University of Maragheh, Maragheh, Iran; b Department of Chemistry, Payame Noor University, PO Box 19395-3697, Tehran, Iran; c Department of Physics, Ondokuz Mayıs University, TR-55139, Samsun, Turkey

## Abstract

In the title compound, C_20_H_23_NO, the cyclo­hepta­none ring adopts a twist-chair conformation, with the amino­methyl substituent in an equatorial position. The relative configuration of the two stereocenters is *R*,*R*. In the crystal, mol­ecules are linked by N—H⋯O hydrogen bonds into chains along [100].

## Related literature

For the synthesis of title compound and related compounds, see: Eftekhari-Sis *et al.* (2013 ▶). For the biological activity of *β*-amino ketones, see: Arend *et al.* (1998 ▶); Jadhav *et al.* (2008 ▶); Kalluraya *et al.* (2001 ▶). For information on the Mannich reaction, see, for example: Eftekhari-Sis *et al.* (2006 ▶); Azizi *et al.* (2006 ▶); Cordova (2004 ▶). For the crystal structures of related compounds, see: Eftekhari-Sis *et al.* (2012 ▶); Yuan *et al.* (2007 ▶); Fun *et al.* (2009 ▶). For puckering parameters, see: Evans & Boeyens (1989 ▶).
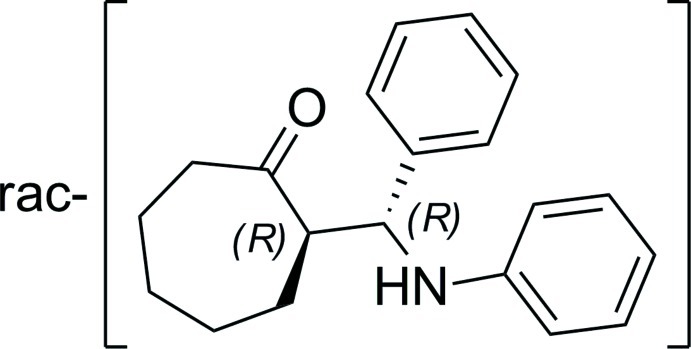


## Experimental

### 

#### Crystal data

C_20_H_23_NO
*M* *_r_* = 293.39
Monoclinic, 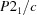

*a* = 5.7534 (4) Å
*b* = 16.1336 (8) Å
*c* = 18.1980 (13) Å
β = 99.371 (6)°
*V* = 1666.65 (19) Å^3^
*Z* = 4
Mo *K*α radiation
μ = 0.07 mm^−1^
*T* = 296 K
0.72 × 0.56 × 0.27 mm

#### Data collection

Stoe IPDS 2 diffractometer
Absorption correction: integration (*X-RED32*; Stoe & Cie, 2002 ▶) *T*_min_ = 0.965, *T*_max_ = 0.985
10757 measured reflections
3449 independent reflections
2170 reflections with *I* > 2σ(*I*)
*R*_int_ = 0.041

#### Refinement

*R*[*F* ^2^ > 2σ(*F* ^2^)] = 0.048
*wR*(*F* ^2^) = 0.136
*S* = 1.01
3449 reflections
203 parameters
H atoms treated by a mixture of independent and constrained refinement
Δρ_max_ = 0.26 e Å^−3^
Δρ_min_ = −0.13 e Å^−3^

### 

Data collection: *X-AREA* (Stoe & Cie, 2002 ▶); cell refinement: *X-AREA*; data reduction: *X-RED32* (Stoe & Cie, 2002 ▶); program(s) used to solve structure: *SHELXS97* (Sheldrick, 2008 ▶); program(s) used to refine structure: *SHELXL97* (Sheldrick, 2008) ▶; molecular graphics: *ORTEP-3 for Windows* (Farrugia, 2012 ▶); software used to prepare material for publication: *WinGX* (Farrugia, 2012 ▶).

## Supplementary Material

Crystal structure: contains datablock(s) I, global. DOI: 10.1107/S1600536812050659/fy2069sup1.cif

Structure factors: contains datablock(s) I. DOI: 10.1107/S1600536812050659/fy2069Isup2.hkl

Supplementary material file. DOI: 10.1107/S1600536812050659/fy2069Isup3.mol

Supplementary material file. DOI: 10.1107/S1600536812050659/fy2069Isup4.cml

Additional supplementary materials:  crystallographic information; 3D view; checkCIF report

## Figures and Tables

**Table 1:** Hydrogen-bond geometry (Å, °)

*D*—H⋯*A*	*D*—H	H⋯*A*	*D*⋯*A*	*D*—H⋯*A*
N1—H1*A*⋯O1^i^	0.879 (19)	2.23 (2)	3.065 (2)	158.4 (16)

Symmetry code: (i) 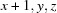.

**Fig. 1.**
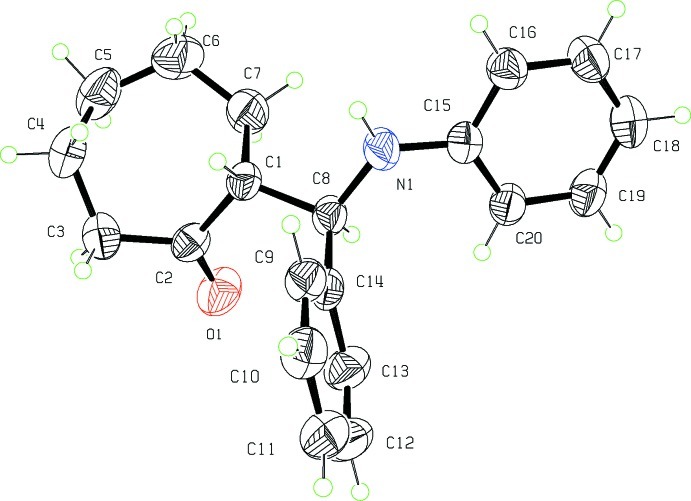
The structure of title compound, showing 40% probability displacement ellipsoids and the atom numbering scheme.

**Fig. 2.**
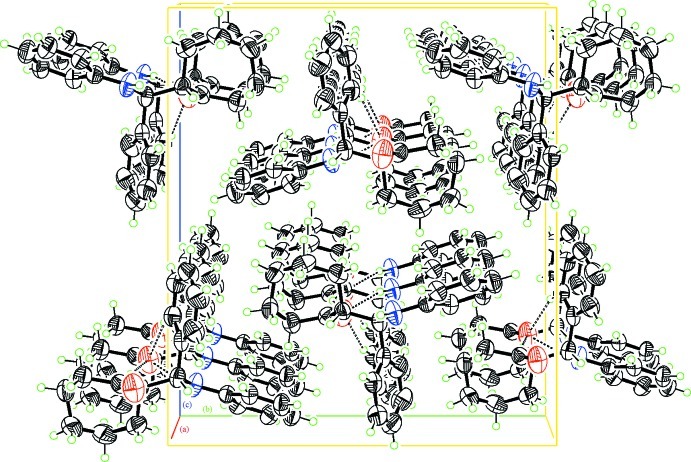
The unit cell contents of title compound.

**Fig. 3.**
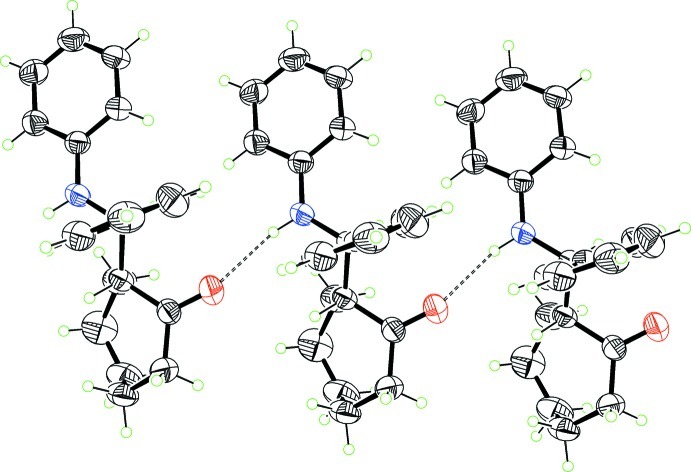
The linking of molecules by N—H···O hydrogen bonds into infinite one-dimensional chains. Hydrogen bonds are shown as dashed lines.

## supplementary crystallographic information

### Comment

Mannich reaction (Eftekhari-Sis *et al.*, 2006; Azizi *et al.*, 2006; Cordova, 2004) is one of the most important basic reactions in organic chemistry for its use in natural product and pharmaceutical syntheses due to formation of C—C and C—N bonds, simultaneously, to furnish β-amino ketones, which exhibit biological activity such as anti-inflammatory (Jadhav *et al.*, 2008) and antimicrobial (Kalluraya *et al.*, 2001) activities. We have synthesized the title compound and report its structure here, Fig 1. The cycloheptanone ring adopts twist-chair conformation, with puckering parameters of Q(2) = 0.539 (2) Å, Φ(2) = 41.7 (2)° and Q(3) = 0.644 (2) Å, Φ(3) = 319.2 (2)° (Evans & Boeyens, 1989). The aminomethyl moiety is positioned equatorially on the ring at C1.

### Experimental

The title compound was obtained by adding of 0.04 g of Laponite-HPMC nano composite (Eftekhari-Sis *et al.*, 2013) to a mixture of 0.5 mmol of benzaldehyde, 0.5 mmol of aniline and 3 equiv. of cycloheptanone and stirring at room temperature for 24 h. After completion of the reaction, 5 ml EtOH was added and the catalyst was removed by filtration. The filtrate was concentrated under reduced pressure. The obtained crude product was recrystallized from EtOH to afford the title compound in 60% yield. Colorless crystals suitable for crystal structure determination were grown from 96% EtOH.

### Refinement

Carbon bound H atoms were positioned geometrically, with C—H=0.93, 0.97, and 0.98 Å for aromatic, methylene and methine H, and constrained to ride on their parent atoms, with *U*_iso_(H) = 1.2Ueq(C). The nitrogen H atoms were located from the difference Fourier map and allowed to refine freely.

### Crystal data

**Table:**

C_20_H_23_NO	*F*(000) = 632
*M**_r_* = 293.39	*D*_x_ = 1.169 Mg m^−^^3^
Monoclinic, *P*2_1_/*c*	Mo *K*α radiation, λ = 0.71073 Å
Hall symbol: -P 2ybc	Cell parameters from 10757 reflections
*a* = 5.7534 (4) Å	θ = 1.7–28.0°
*b* = 16.1336 (8) Å	µ = 0.07 mm^−^^1^
*c* = 18.1980 (13) Å	*T* = 296 K
β = 99.371 (6)°	Prism, colourless
*V* = 1666.65 (19) Å^3^	0.72 × 0.56 × 0.27 mm
*Z* = 4	

### Data collection

**Table:**

Stoe IPDS 2 diffractometer	3449 independent reflections
Radiation source: fine-focus sealed tube	2170 reflections with *I* > 2σ(*I*)
Graphite monochromator	*R*_int_ = 0.041
rotation method scans	θ_max_ = 26.5°, θ_min_ = 1.7°
Absorption correction: integration (*X-RED32*; Stoe & Cie, 2002)	*h* = −7→7
*T*_min_ = 0.965, *T*_max_ = 0.985	*k* = −20→20
10757 measured reflections	*l* = −22→18

### Refinement

**Table:**

Refinement on *F*^2^	Primary atom site location: structure-invariant direct methods
Least-squares matrix: full	Secondary atom site location: difference Fourier map
*R*[*F*^2^ > 2σ(*F*^2^)] = 0.048	Hydrogen site location: inferred from neighbouring sites
*wR*(*F*^2^) = 0.136	H atoms treated by a mixture of independent and constrained refinement
*S* = 1.01	*w* = 1/[σ^2^(*F*_o_^2^) + (0.0722*P*)^2^] where *P* = (*F*_o_^2^ + 2*F*_c_^2^)/3
3449 reflections	(Δ/σ)_max_ < 0.001
203 parameters	Δρ_max_ = 0.26 e Å^−^^3^
0 restraints	Δρ_min_ = −0.13 e Å^−^^3^

### Special details

**Table:**

Geometry. All e.s.d.'s (except the e.s.d. in the dihedral angle between two l.s. planes) are estimated using the full covariance matrix. The cell e.s.d.'s are taken into account individually in the estimation of e.s.d.'s in distances, angles and torsion angles; correlations between e.s.d.'s in cell parameters are only used when they are defined by crystal symmetry. An approximate (isotropic) treatment of cell e.s.d.'s is used for estimating e.s.d.'s involving l.s. planes.
Refinement. Refinement of *F*^2^ against ALL reflections. The weighted *R*-factor *wR* and goodness of fit *S* are based on *F*^2^, conventional *R*-factors *R* are based on *F*, with *F* set to zero for negative *F*^2^. The threshold expression of *F*^2^ > σ(*F*^2^) is used only for calculating *R*-factors(gt) *etc*. and is not relevant to the choice of reflections for refinement. *R*-factors based on *F*^2^ are statistically about twice as large as those based on *F*, and *R*- factors based on ALL data will be even larger.

### Fractional atomic coordinates and isotropic or equivalent isotropic displacement parameters (Å^2^)

**Table:**

	*x*	*y*	*z*	*U*_iso_*/*U*_eq_	
C1	0.4026 (3)	0.45073 (9)	0.32943 (9)	0.0508 (4)	
H1	0.5213	0.4211	0.3065	0.061*	
C2	0.1676 (3)	0.41004 (10)	0.30531 (10)	0.0545 (4)	
C3	0.1590 (3)	0.31909 (11)	0.28983 (11)	0.0680 (5)	
H3A	0.1590	0.3111	0.2370	0.082*	
H3B	0.0108	0.2978	0.3008	0.082*	
C4	0.3563 (4)	0.26786 (11)	0.33231 (12)	0.0750 (6)	
H4A	0.3205	0.2097	0.3229	0.090*	
H4B	0.5002	0.2801	0.3131	0.090*	
C5	0.3998 (5)	0.28183 (13)	0.41489 (13)	0.0919 (7)	
H5A	0.2488	0.2880	0.4315	0.110*	
H5B	0.4747	0.2328	0.4388	0.110*	
C6	0.5488 (5)	0.35543 (15)	0.44101 (12)	0.0922 (7)	
H6A	0.5733	0.3557	0.4950	0.111*	
H6B	0.7017	0.3476	0.4261	0.111*	
C7	0.4594 (4)	0.43901 (13)	0.41458 (11)	0.0786 (6)	
H7A	0.5761	0.4800	0.4346	0.094*	
H7B	0.3177	0.4505	0.4353	0.094*	
C8	0.4030 (3)	0.54291 (9)	0.30610 (10)	0.0533 (4)	
H8	0.2812	0.5716	0.3283	0.064*	
C9	0.5035 (3)	0.52774 (10)	0.17700 (11)	0.0606 (5)	
H9	0.6439	0.5026	0.1982	0.073*	
C10	0.4556 (4)	0.53841 (12)	0.10111 (12)	0.0726 (5)	
H10	0.5642	0.5210	0.0717	0.087*	
C11	0.2490 (4)	0.57456 (13)	0.06854 (13)	0.0801 (6)	
H11	0.2159	0.5810	0.0171	0.096*	
C12	0.0910 (4)	0.60127 (14)	0.11243 (14)	0.0850 (7)	
H12	−0.0489	0.6264	0.0907	0.102*	
C13	0.1397 (3)	0.59089 (12)	0.18865 (12)	0.0713 (5)	
H13	0.0314	0.6093	0.2178	0.086*	
C14	0.3459 (3)	0.55379 (9)	0.22262 (10)	0.0530 (4)	
C15	0.6636 (3)	0.65940 (9)	0.36011 (9)	0.0525 (4)	
C16	0.8846 (3)	0.68313 (11)	0.39812 (10)	0.0604 (4)	
H16	1.0051	0.6441	0.4065	0.073*	
C17	0.9275 (4)	0.76247 (12)	0.42320 (12)	0.0706 (5)	
H17	1.0764	0.7766	0.4481	0.085*	
C18	0.7531 (4)	0.82152 (12)	0.41206 (12)	0.0760 (6)	
H18	0.7823	0.8754	0.4293	0.091*	
C19	0.5343 (4)	0.79933 (11)	0.37481 (12)	0.0688 (5)	
H19	0.4152	0.8389	0.3671	0.083*	
C20	0.4877 (3)	0.71963 (10)	0.34864 (10)	0.0587 (4)	
H20	0.3387	0.7062	0.3233	0.070*	
N1	0.6284 (3)	0.57845 (9)	0.33722 (10)	0.0661 (5)	
O1	−0.0127 (2)	0.44979 (8)	0.30255 (10)	0.0854 (5)	
H1A	0.751 (3)	0.5457 (11)	0.3392 (10)	0.064 (5)*	

### Atomic displacement parameters (Å^2^)

**Table:**

	*U*^11^	*U*^22^	*U*^33^	*U*^12^	*U*^13^	*U*^23^
C1	0.0554 (9)	0.0428 (8)	0.0545 (10)	0.0063 (7)	0.0095 (7)	−0.0041 (7)
C2	0.0518 (9)	0.0529 (9)	0.0604 (11)	0.0067 (8)	0.0142 (8)	0.0069 (8)
C3	0.0697 (11)	0.0561 (10)	0.0797 (13)	−0.0070 (9)	0.0166 (9)	−0.0084 (9)
C4	0.0957 (15)	0.0449 (9)	0.0878 (15)	0.0060 (10)	0.0253 (11)	0.0033 (9)
C5	0.132 (2)	0.0664 (13)	0.0802 (16)	0.0179 (13)	0.0271 (14)	0.0195 (11)
C6	0.1148 (18)	0.0980 (17)	0.0584 (13)	0.0108 (15)	−0.0023 (12)	0.0184 (11)
C7	0.1024 (16)	0.0695 (12)	0.0605 (12)	0.0015 (11)	0.0029 (11)	−0.0085 (10)
C8	0.0482 (9)	0.0410 (8)	0.0709 (11)	0.0037 (7)	0.0104 (8)	−0.0043 (7)
C9	0.0577 (10)	0.0510 (9)	0.0719 (13)	0.0038 (8)	0.0067 (9)	−0.0009 (8)
C10	0.0826 (14)	0.0633 (12)	0.0738 (14)	0.0020 (10)	0.0182 (11)	−0.0008 (9)
C11	0.0962 (16)	0.0693 (12)	0.0719 (13)	0.0007 (12)	0.0054 (12)	0.0129 (10)
C12	0.0760 (14)	0.0830 (14)	0.0903 (17)	0.0155 (11)	−0.0031 (12)	0.0220 (12)
C13	0.0606 (11)	0.0674 (11)	0.0857 (15)	0.0125 (9)	0.0110 (10)	0.0134 (10)
C14	0.0509 (9)	0.0348 (7)	0.0730 (12)	−0.0021 (7)	0.0092 (8)	0.0022 (7)
C15	0.0585 (10)	0.0456 (8)	0.0551 (10)	−0.0011 (7)	0.0142 (7)	−0.0021 (7)
C16	0.0588 (10)	0.0578 (10)	0.0639 (11)	0.0009 (8)	0.0076 (8)	−0.0071 (8)
C17	0.0700 (12)	0.0648 (11)	0.0757 (13)	−0.0106 (10)	0.0076 (10)	−0.0152 (9)
C18	0.0901 (14)	0.0522 (10)	0.0873 (15)	−0.0114 (10)	0.0198 (11)	−0.0138 (10)
C19	0.0754 (12)	0.0462 (9)	0.0876 (14)	0.0064 (9)	0.0215 (10)	0.0000 (9)
C20	0.0591 (10)	0.0469 (9)	0.0707 (12)	0.0001 (8)	0.0122 (8)	0.0007 (8)
N1	0.0520 (9)	0.0463 (8)	0.0961 (13)	0.0057 (7)	0.0010 (8)	−0.0161 (7)
O1	0.0558 (8)	0.0696 (9)	0.1328 (14)	0.0105 (7)	0.0216 (8)	0.0106 (8)

### Geometric parameters (Å, º)

**Table:**

C1—C2	1.502 (2)	C9—C14	1.390 (2)
C1—C7	1.542 (2)	C9—H9	0.9300
C1—C8	1.547 (2)	C10—C11	1.369 (3)
C1—H1	0.9800	C10—H10	0.9300
C2—O1	1.214 (2)	C11—C12	1.373 (3)
C2—C3	1.493 (2)	C11—H11	0.9300
C3—C4	1.511 (3)	C12—C13	1.380 (3)
C3—H3A	0.9700	C12—H12	0.9300
C3—H3B	0.9700	C13—C14	1.382 (2)
C4—C5	1.500 (3)	C13—H13	0.9300
C4—H4A	0.9700	C15—N1	1.376 (2)
C4—H4B	0.9700	C15—C20	1.394 (2)
C5—C6	1.496 (3)	C15—C16	1.399 (2)
C5—H5A	0.9700	C16—C17	1.368 (2)
C5—H5B	0.9700	C16—H16	0.9300
C6—C7	1.495 (3)	C17—C18	1.375 (3)
C6—H6A	0.9700	C17—H17	0.9300
C6—H6B	0.9700	C18—C19	1.376 (3)
C7—H7A	0.9700	C18—H18	0.9300
C7—H7B	0.9700	C19—C20	1.382 (2)
C8—N1	1.446 (2)	C19—H19	0.9300
C8—C14	1.511 (2)	C20—H20	0.9300
C8—H8	0.9800	N1—H1A	0.879 (19)
C9—C10	1.374 (3)		
			
C2—C1—C7	105.86 (14)	N1—C8—H8	107.9
C2—C1—C8	112.43 (13)	C14—C8—H8	107.9
C7—C1—C8	112.58 (14)	C1—C8—H8	107.9
C2—C1—H1	108.6	C10—C9—C14	121.25 (17)
C7—C1—H1	108.6	C10—C9—H9	119.4
C8—C1—H1	108.6	C14—C9—H9	119.4
O1—C2—C3	120.63 (16)	C11—C10—C9	120.4 (2)
O1—C2—C1	120.23 (15)	C11—C10—H10	119.8
C3—C2—C1	119.02 (14)	C9—C10—H10	119.8
C2—C3—C4	116.21 (16)	C10—C11—C12	119.5 (2)
C2—C3—H3A	108.2	C10—C11—H11	120.3
C4—C3—H3A	108.2	C12—C11—H11	120.3
C2—C3—H3B	108.2	C11—C12—C13	120.1 (2)
C4—C3—H3B	108.2	C11—C12—H12	120.0
H3A—C3—H3B	107.4	C13—C12—H12	120.0
C5—C4—C3	114.78 (17)	C12—C13—C14	121.4 (2)
C5—C4—H4A	108.6	C12—C13—H13	119.3
C3—C4—H4A	108.6	C14—C13—H13	119.3
C5—C4—H4B	108.6	C13—C14—C9	117.37 (18)
C3—C4—H4B	108.6	C13—C14—C8	122.03 (16)
H4A—C4—H4B	107.5	C9—C14—C8	120.59 (15)
C6—C5—C4	115.53 (18)	N1—C15—C20	123.29 (16)
C6—C5—H5A	108.4	N1—C15—C16	119.13 (15)
C4—C5—H5A	108.4	C20—C15—C16	117.57 (15)
C6—C5—H5B	108.4	C17—C16—C15	121.42 (17)
C4—C5—H5B	108.4	C17—C16—H16	119.3
H5A—C5—H5B	107.5	C15—C16—H16	119.3
C7—C6—C5	117.68 (19)	C16—C17—C18	120.77 (18)
C7—C6—H6A	107.9	C16—C17—H17	119.6
C5—C6—H6A	107.9	C18—C17—H17	119.6
C7—C6—H6B	107.9	C17—C18—C19	118.66 (17)
C5—C6—H6B	107.9	C17—C18—H18	120.7
H6A—C6—H6B	107.2	C19—C18—H18	120.7
C6—C7—C1	116.06 (17)	C18—C19—C20	121.50 (18)
C6—C7—H7A	108.3	C18—C19—H19	119.3
C1—C7—H7A	108.3	C20—C19—H19	119.3
C6—C7—H7B	108.3	C19—C20—C15	120.08 (17)
C1—C7—H7B	108.3	C19—C20—H20	120.0
H7A—C7—H7B	107.4	C15—C20—H20	120.0
N1—C8—C14	112.45 (14)	C15—N1—C8	124.99 (15)
N1—C8—C1	108.37 (13)	C15—N1—H1A	118.8 (12)
C14—C8—C1	112.20 (13)	C8—N1—H1A	116.2 (11)
			
C7—C1—C2—O1	90.59 (19)	C12—C13—C14—C9	0.4 (3)
C8—C1—C2—O1	−32.7 (2)	C12—C13—C14—C8	179.25 (17)
C7—C1—C2—C3	−85.34 (18)	C10—C9—C14—C13	−0.1 (2)
C8—C1—C2—C3	151.35 (16)	C10—C9—C14—C8	−178.96 (15)
O1—C2—C3—C4	−147.48 (19)	N1—C8—C14—C13	−125.11 (17)
C1—C2—C3—C4	28.4 (2)	C1—C8—C14—C13	112.42 (17)
C2—C3—C4—C5	50.6 (2)	N1—C8—C14—C9	53.71 (18)
C3—C4—C5—C6	−82.4 (3)	C1—C8—C14—C9	−68.77 (18)
C4—C5—C6—C7	61.6 (3)	N1—C15—C16—C17	−178.84 (17)
C5—C6—C7—C1	−56.2 (3)	C20—C15—C16—C17	0.0 (3)
C2—C1—C7—C6	78.4 (2)	C15—C16—C17—C18	0.3 (3)
C8—C1—C7—C6	−158.42 (19)	C16—C17—C18—C19	−0.3 (3)
C2—C1—C8—N1	175.01 (14)	C17—C18—C19—C20	−0.1 (3)
C7—C1—C8—N1	55.55 (19)	C18—C19—C20—C15	0.4 (3)
C2—C1—C8—C14	−60.23 (18)	N1—C15—C20—C19	178.42 (18)
C7—C1—C8—C14	−179.69 (15)	C16—C15—C20—C19	−0.4 (3)
C14—C9—C10—C11	−0.6 (3)	C20—C15—N1—C8	−7.0 (3)
C9—C10—C11—C12	1.0 (3)	C16—C15—N1—C8	171.72 (16)
C10—C11—C12—C13	−0.7 (3)	C14—C8—N1—C15	87.9 (2)
C11—C12—C13—C14	0.0 (3)	C1—C8—N1—C15	−147.48 (17)

### Hydrogen-bond geometry (Å, º)

**Table:**

*D*—H···*A*	*D*—H	H···*A*	*D*···*A*	*D*—H···*A*
N1—H1*A*···O1^i^	0.879 (19)	2.23 (2)	3.065 (2)	158.4 (16)

Symmetry code: (i) *x*+1, *y*, *z*.
